# The Upconversion Luminescence of Er^3+^/Yb^3+^/Nd^3+^ Triply-Doped β-NaYF_4_ Nanocrystals under 808-nm Excitation

**DOI:** 10.3390/ma7117289

**Published:** 2014-11-04

**Authors:** Lijiao Tian, Zheng Xu, Suling Zhao, Yue Cui, Zhiqin Liang, Junjie Zhang, Xurong Xu

**Affiliations:** Key Laboratory of Luminescence and Optical Information, Beijing Jiaotong University, Ministry of Education, Beijing 100044, China; E-Mails: 12121711@bjtu.edu.cn (L.T.); slzhao@bjtu.edu.cn (S.Z.); 11118404@bjtu.edu.cn (Y.C.); 09273041@bjtu.edu.cn (Z.L.); 12121659@bjtu.edu.cn (J.Z.); xrxu@bjtu.edu.cn (X.X.)

**Keywords:** upconversion, nanoparticles, Nd^3+^ concentration, mechanism

## Abstract

In this paper, Nd^3+^–Yb^3+^–Er^3+^-doped β-NaYF_4_ nanocrystals with different Nd^3+^ concentrations are synthesized, and the luminescence properties of the upconversion nanoparticles (UCNPs) have been studied under 808-nm excitation for sensitive biological applications. The upconversion luminescence spectra of NaYF_4_ nanoparticles with different dopants under 808-nm excitation proves that the Nd^3+^ ion can absorb the photons effectively, and the Yb^3+^ ion can play the role of an energy-transfer bridging ion between the Nd^3+^ ion and Er^3+^ ion. To investigate the effect of the Nd^3+^ ion, the decay curves of the ^4^S_3/2_ → ^4^I_15/2_ transition at 540 nm are measured and analyzed. The NaYF_4_: 20% Yb^3+^, 2% Er^3+^, 0.5% Nd^3+^ nanocrystals have the highest emission intensity among all samples under 808-nm excitation. The UC (upconversion) mechanism under 808-nm excitation is discussed in terms of the experimental results.

## 1. Introduction

In recent years, lanthanide-doped upconversion nanoparticles (UCNPs) have attracted extensive research interest [1,2,3,4]. Benefiting from the effective penetration depth of near-infrared (NIR) photons in biological tissues and minimized auto-fluorescence background, UCNPs are widely used for a variety of biological applications [5,6,7,8], such as labeling, imaging and photodynamic therapy. In most cases, these UCNPs are doped simultaneously with sensitizer ions (for example, Yb^3+^) and activator ions (for example, Er^3+^, Tm^3+^,or Ho^3+^) [9]. The sensitizer ions absorb NIR photons and then transfer the energy to the activator ions [10]. The energy transfer (ET) will excite the activators to their higher excited states and eventually lead to the radiation of higher-energy photons [10]. The hexagonal NaYF_4_, due to its low phonon energy and high chemical stability, has been confirmed as one of the most efficient UCNP host [11].

**Figure 1:**
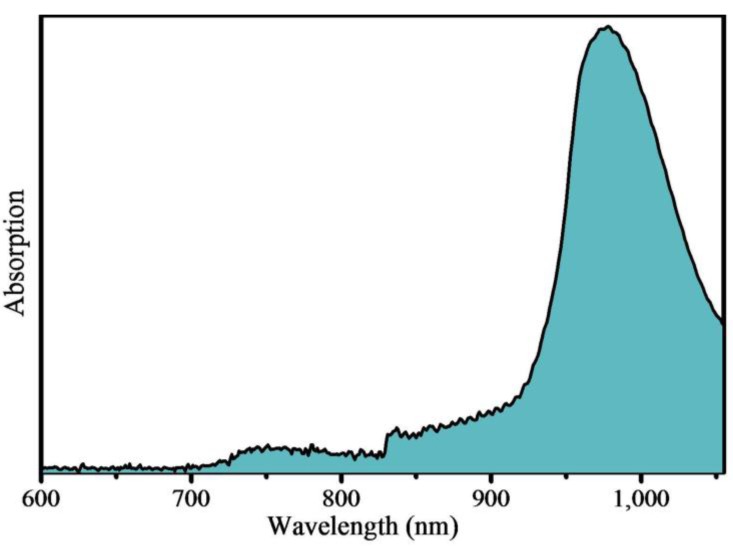
Absorption of water in the NIR (near-infrared).

However, the Yb^3+^ ion-sensitized UC (upconversion) process is challenging for *in vivo* biological applications, because of the narrow band absorption of Yb^3+^ ions, which has only one excited state (^2^F_5/2_) corresponding to the absorption around 980 nm from ^2^F_7/2_ to ^2^F_5/2_. Water, the most concentrated and significant NIR (near-infrared) absorber in biological tissues, also has high absorption around this band, as shown in Figure 1. Therefore, using a continuous laser of 980 nm to trigger the UC process would lead to the risk of overheating and even may induce cell and tissue damage [12,13]. It is very urgent to use another NIR excitation laser corresponding to a low absorption region of water to realize UC processes for biological applications of UCNPs.

According to Figure 1, water has a low absorption (Figure 1) around 808 nm, in contrast with that at 980 nm. Among rare earth ions, the Nd^3+^ ion has relatively strong absorption around 808 nm, corresponding to the transition from ^4^I_9/2_ to ^4^F_5/2_. Herein, we introduce Nd^3+^ ions into NaYF_4_: 20% Yb^3+^, 2% Er^3+^ UCNPs, which has been reported as one of the most efficient UCP (upconversion particle) materials [14]. Thus, Nd^3+^ ions will be a new NIR absorber and sensitizer to address the issue of NIR laser-induced tissue damage by using excitation at 808 nm. Interestingly, the Yb^3+^ ion can play the role of an energy-transfer bridging ion between an energy donor (Nd^3+^) ion and an energy acceptor ion (Er^3+^) under 808-nm excitation [15]. Consequently, the laser-induced overheating effect under 808-nm excitation, especially for biological tissues, is expected to be greatly minimized. Meanwhile, the Nd^3+^ ion has a large absorption cross-section around 800 nm [16], which is expected to enhance the pumping efficiency of the Er^3+^ ions.

## 2. Results and Discussion

**Figure 2:**
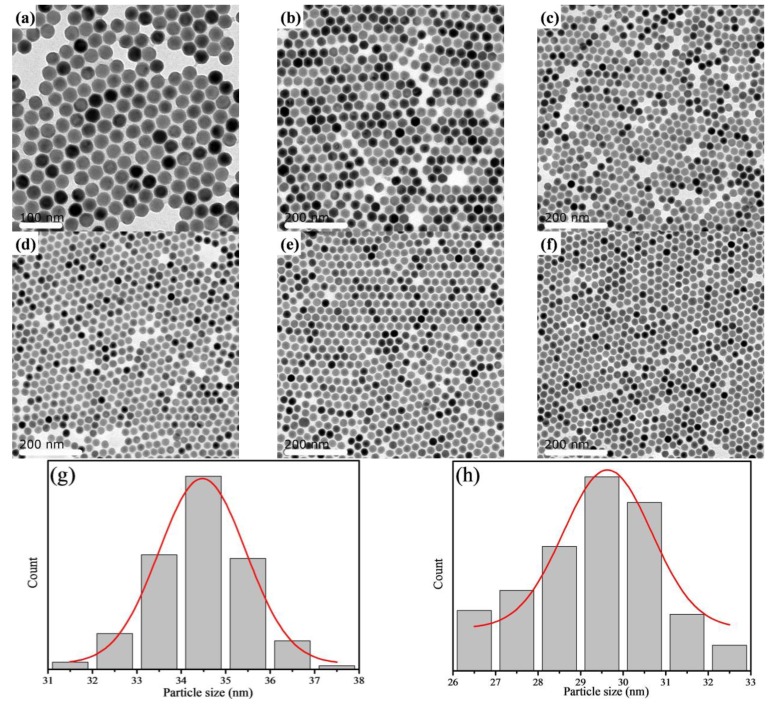

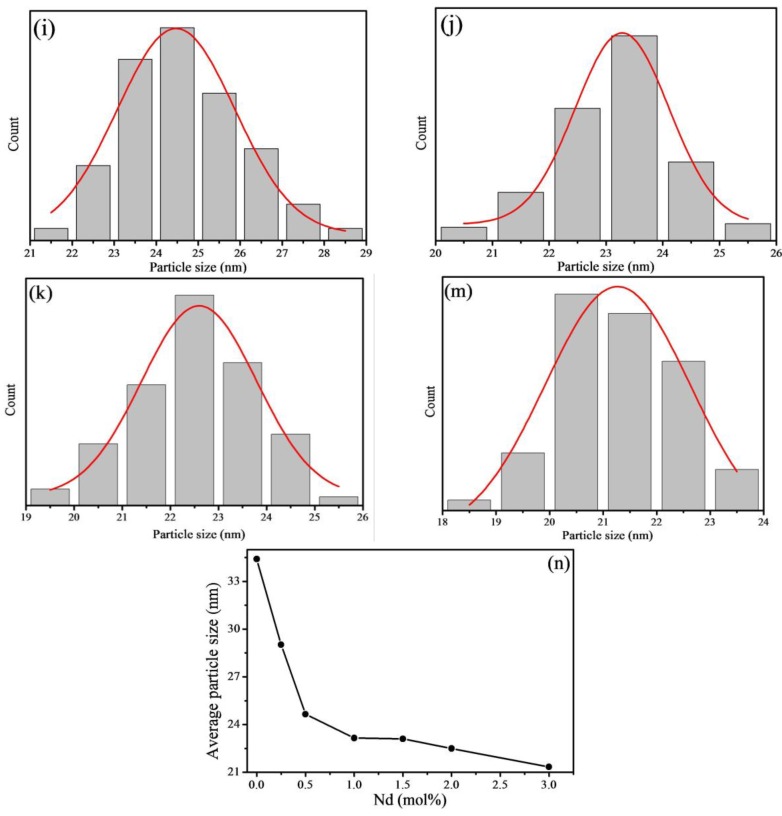
TEM (transmission electron microscopy) image and the size distribution of NaYF_4_: Yb/Er/Nd (20/2/*X* mol%) nanoparticles: (**a**,**g**) *X* = 0; (**b**,**h**) *X* = 0.25; (**c**,**i**) *X* = 0.5; (**d**,**j**) *X* = 1.0; (**e**,**k**) *X* = 2.0; (**f**,**m**) *X* = 3.0; (**n**) average nanoparticle diameter of NaYF_4_: Yb/Er/Nd (20/2/*X* mol%).

As shown in the transmission electron microscopy (TEM) images (Figure 2a–f), all of the Nd^3+^–Yb^3+^–Er^3+^-doped UCNPs have a uniform morphology with an average diameter between 20 nm and 35 nm. The calculated average particle size for different amounts of Nd^3+^ ions are shown in Figure 2n. As the concentration of Nd^3+^ ions increases, the average particle size decreases from ~34 nm to ~21 nm.

**Figure 3:**
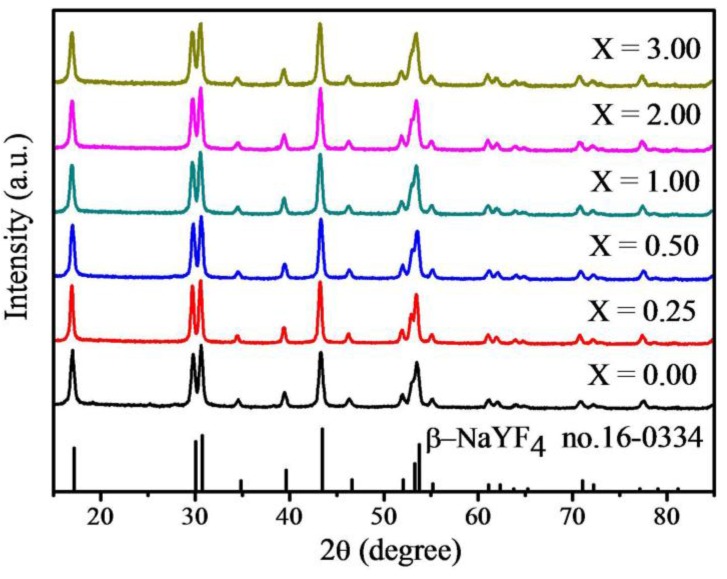
XRD pattern of the as-synthesized NaYF_4_: Yb/Er/Nd (20/2/*X* mol%) nanoparticles.

With the same concentration of Yb^3+^ ions and Er^3+^ ions, parts of the Y^3+^ ions (*r* = 1.159 Å) [17] were replaced by Nd^3+^ ions (*r* = 1.249 Å) [17]. This confirms that doping of the lanthanide ion with a size larger than the Y^3+^ ion in NaYF_4_ host lattices should result in the formation of smaller nanoparticles. The X-ray diffraction (XRD) patterns of samples in Figure 3 indicate that all of the samples crystallize into the hexagonal NaYF_4_, which accords basically with the standard X-ray diffraction JCPDS (joint committee on powder diffraction standards) 16-0334.

**Figure 4:**
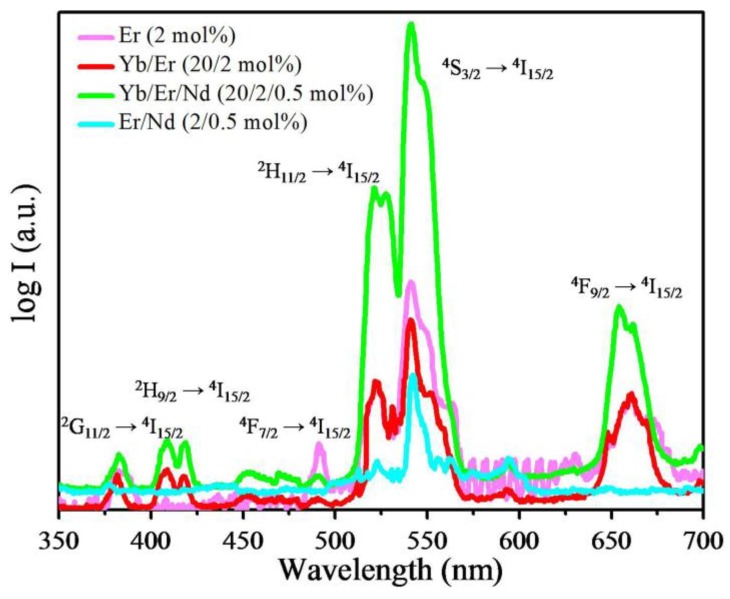
Upconversion emission spectra using a log scale for the emission intensity of NaYF_4_: Er (2 mol%), NaYF_4_: Yb/Er (20/2 mol%), NaYF_4_: Yb/Er/Nd (20/2/0.5 mol%) and NaYF_4_: Er/Nd (2/0.5 mol%) nanoparticles under 808-nm excitation.

Figure 4 shows the upconversion luminescence spectra of NaYF_4_ nanoparticles with different dopants. All four samples produce upconversion photoluminescence (PL) under excitation at 808 nm using a power density of 3 W/cm^2^. As shown in Figure 4, four major sensitized UC emission bands centered at 410 nm, 520 nm, 540 nm and 655 nm, which correspond to the transitions of Er^3+^ ions: ^2^H_9/2_ → ^4^I_15/2_, ^2^H_11/2_ → ^4^I_15/2_, ^4^S_3/2_ → ^4^I_15/2_ and ^4^F_9/2_ → ^4^I_15/2_, respectively, are observed. There are emissions of Er^3+^ ions even on the premise that NaYF_4_ host lattices are doped only with Er^3+^ ions. This means that the UC emission of Er^3+^ ions mostly results from the limited excited state absorption of Er^3+^ ions. The Yb^3+^ ions can hardly absorb the photons at 808 nm. Therefore, doping with Yb^3+^/Er^3+^ can only weaken the emission of Er^3+^ ions by an energy back transfer process from Er^3+^ ions to Yb^3+^ ions. Moreover, it can be seen that the emission of Er^3+^ ions is also weakened after doping with Nd^3+^/Er^3+^, although Nd^3+^ ions can absorb the photons at 808 nm effectively. This shows that Nd^3+^ ions cannot transfer their energy to Er^3+^ ions efficiently. The excited Nd^3+^ ions lose their energy to the other states radiatively or relax to their ground state non-radiatively. The intensity of upconversion luminescence of NaYF_4_: Nd^3+^/Er^3+^ is lower than that of NaYF_4_: Yb^3+^/Er^3+^, which shows that the energy back transfer Er^3+^ → Nd^3+^ is much more efficient than Er^3+^ → Yb^3+^, even when the Nd^3+^ concentration (0.5 mol%) is much lower than the Yb^3+^ concentration (20 mol%). Furthermore, due to codoping with Yb^3+^ ions, the luminescence intensity of sample NaYF_4_: Nd^3+^/Yb^3+^/Er^3+^ is much stronger than that of NaYF_4_: Nd^3+^Er^3+^. This shows that Yb^3+^ ions can act as the bridging center to prompt energy transfer from Nd^3+^ ions to Er^3+^ ions under 808-nm excitation.

**Figure 5:**
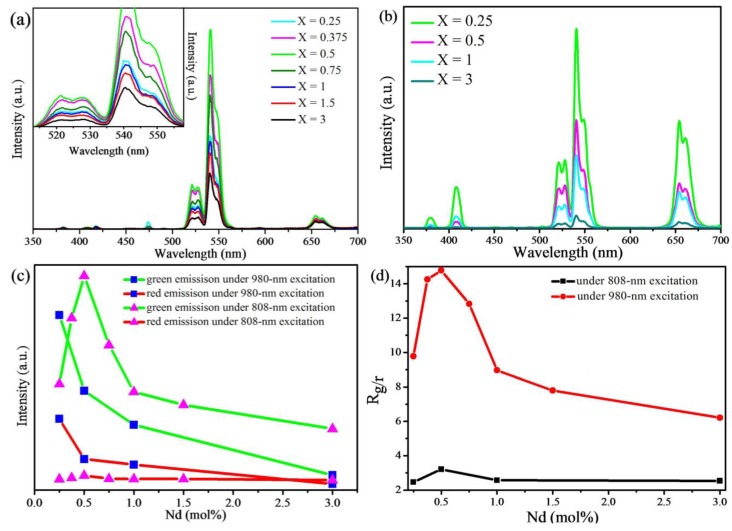
Upconversion emission spectra of the NaYF_4_: Yb/Er/Nd (20/2/*X* mol%) nanoparticles under (**a**) 808-nm excitation and (**b**) 980-nm excitation. The inserted image in (**a**) is the magnified green emissions; (**c**,**d**) the dependence of green and red emission intensity on Nd^3+^ concentration and the dependence of *R*_g/r_ on Nd^3+^ concentration of the NaYF_4_: Yb/Er/Nd (20/2/*X* mol%) nanoparticles under 808-nm and 980-nm excitation.

As shown in Figure 5a,b, four sensitized UC emission bands centered at 410, 520, 540 and 655 nm, which correspond to the transitions of Er^3+^ ions: ^2^H_9/2_ → ^4^I_15/2_, ^2^H_11/2_ → ^4^I_15/2_, ^4^S_3/2_ → ^4^I_15/2_ and ^4^F_9/2_ → ^4^I_15/2_, respectively, are observed both under 808- and 980-nm excitation using a power density of 2.5 and 250 mW/cm^2^, respectively. In this paper, we discuss only the green and red emission bands. Figure 5c,d shows that the intensity ratio of green to red emission (*R*_g/r_) is higher under 808-nm excitation than the ratio under 980-nm excitation. As the Nd^3+^ concentration increases, both the intensity ratios of green to red emission under 980- and 808-nm excitation increase to a maximum and then decrease gradually. Introducing Nd^3+^ ions with different amounts into NaYF_4_: Yb^3+^/Er^3+^ has an effect on the non-radiative energy back transfer from Er^3+^ ions to Nd^3+^ ions and then influences the cross-relaxation process of Er^3+^ ions, which dominate the green-to-red emission ratio [18]. Therefore, it is concluded that the Nd^3+^ ions with a certain concentration are effective at originating the green emission of Er^3+^ ions. In addition, the high intensity ratio of green to red emission under 808-nm excitation shows that 808-nm excitation is favored over the green emission of Er^3+^ ions for biomarkers.

The UC luminescence spectra presented in Figure 5a reveal the effect of Nd^3+^ concentration on the emission intensity of Er^3+^ under 808-nm excitation. The intensity of Er^3+^ emissions is enhanced when the concentration of Nd^3+^ ions ranges from 0.0 to 0.5 mol%, then decreases when the Nd^3+^ concentration exceeds 0.5 mol%. It can be concluded that co-doping with a low content of Nd^3+^ ions will promote the upconversion emission of Er^3+^ ions, and the upconversion intensity of Er^3+^ ions depends greatly on the concentration of Nd^3+^ ions. Therefore, the energy transfer from Nd^3+^ ions to Er^3+^ ions is the main mechanism to produce the upconversion emission of Er^3+^ ions. When the concentration of Nd^3+^ is more than 0.5 mol%, the upconversion intensity of the Er^3+^ ions decreases greatly. The more Nd^3+^ ions there are in the UCNPs, the shorter the distance is between the adjacent ions. The energy back transfer from Er^3+^ ions to Nd^3+^ ions and the non-radiative cross-relaxation (CR) between Nd^3+^ ions become particularly active once the concentration of Nd^3+^ ions is higher than 0.5 mol%. As a result, some Er^3+^ ions are de-excited by a resonant cross-relaxation energy transfer to Nd^3+^ ions [19], which leads to a decrease in the upconversion intensity of the 520-, 540- and 645-nm emissions of Er^3+^ ions. Furthermore, the distortion of the local symmetry around the Er^3+^ ions is another significant reason for enhancing emission. When the concentration of Nd^3+^ ions increases to over 0.5 mol%, more and more Nd^3+^ ions occupy interstitial sites, which will cause the increasing defect centers. On the other hand, an Er^3+^ ion may be surrounded by more than one Nd^3+^ ion with the increase of Nd^3+^ concentration. This renders the crystal field around Er^3+^ more symmetric again. The UC emission is thus reduced. Therefore, for the sake of using the 808-nm excitation for biological applications of UCNPs, Nd^3+^ ions with a concentration of about 0.5 mol% are necessary to be doped in NaYF_4_: 20% Yb^3+^, 2% Er^3+^ UCNPs.

In order to study the energy back transfer from Er^3+^ ions to Nd^3+^ ions, the upconversion spectra of prepared UCNPs under 980-nm excitation were detected. There are only the emissions of Er^3+^ ions in the visible light region. The energy transfer from Yb^3+^ ions to Er^3+^ ions is the main upconversion mechanism under 980-nm excitation. The Nd^3+^ ion makes almost no positive contribution to the upconversion emission of Er^3+^ ions under 980-nm excitation. However, the intensity of the 520-, 540- and 655-nm emissions of Er^3+^ ions decreases with increasing Nd^3+^ concentration, which indicates that the Nd^3+^ ion has a quenching effect on the upconversion emissions of Er^3+^ under 980-nm excitation. There may be an energy transfer from Er^3+^ ions to Nd^3+^ ions. With increasing Nd^3+^ concentration, the energy transfer from Er^3+^ to Nd^3+^ ions becomes more efficient. Some Er^3+^ ions transfer energy to Nd^3+^ ions by three resonant cross-relaxation (CR) transitions:

^4^F_9/2_ (Er^3+^) + ^4^I_9/2_ (Nd^3+^) → ^4^I_15/2_ (Er^3+^) + ^4^F_9/2_ (Nd^3+^) (Δ*E* = −936 cm^−1^)

^4^I_11/2_ (Er^3+^) + ^4^I_9/2_ (Nd^3+^) → ^4^I_13/2_ (Er^3+^) + ^4^I_13/2_ (Nd^3+^) (Δ*E* = +267 cm^−1^)

^4^I_13/2_ (Er^3+^) + ^4^I_9/2_ (Nd^3+^) → ^4^I_15/2_ (Er^3+^) + ^4^I_15/2_ (Nd^3+^) (Δ*E* = −644 cm^−1^)

These three processes depopulate the ^4^F_9/2_, ^4^I_11/2_ and ^4^I_13/2_ states of Er^3+^, respectively. As a result, the luminescence of Er^3+^ is quenched and gradually becomes weak with an increase in the Nd^3+^ ion concentration. To avoid the energy back transfer from emitting ions to the primary sensitizer, the Yb^3+^/Er^3+^-doped core and Nd^3+^/Yb^3+^ co-doped shell should help, since the Nd^3+^ ion is separated from the Er^3+^ ion in such a case. This idea has already been presented in the literature [20,21].

**Figure 6:**
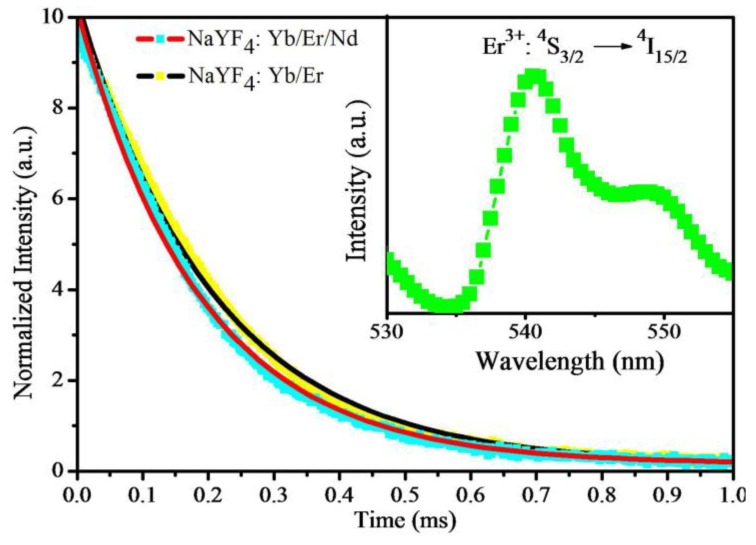
Luminescence decay curves of Er^3+^: ^4^S_3/2_ → ^4^I_15/2_ transition in NaYF_4_: Yb/Er (20/2 mol%) nanoparticles with and without Nd (0.5 mol%) co-doping. The inserted image is the emission spectrum of the ^4^S_3/2_ → ^4^I_15/2_ transition under 980-nm excitation.

In order to study the luminescence kinetics and the mechanism of the upconversion emission, the decay curves of ^4^S_3/2_ → ^4^I_15/2_ (540 nm) for the samples were measured. The normalized decay curves of the ^4^S_3/2_ → ^4^I_15/2_ transition at 540 nm for the NaYF_4_: Yb^3+^/Er^3+^ without and with Nd^3+^ ions under 980-nm excitation are presented in Figure 6. Each of the decay curves of the samples could be fitted to a single exponential function *I*(*t*) = *I*_0_exp(−*t*/τ), where *I* and *I*_0_ are the emission intensities at time *t* and zero, and τ is the lifetime [22]. As shown in Figure 6, the lifetime decreases from 207 to 187 μs after doping with 0.5 mol% Nd^3+^ ions. It can be proven that there occurs an energy back transfer from Er^3+^ ions to Nd^3+^ ions.

The measured rise times are shown in Table 1 with respect to Nd^3+^ concentration. The rise time and fall time of the 808-nm pulse is very short compared with that of the upconversion emissions and is neglected. This shows in Table 1 that the rise time of samples NaYF_4_: Nd^3+^/Yb^3+^/Er^3+^ is longer than that of the sample NaYF_4_: Nd^3+^/Er^3+^ when the concentration of Nd^3+^ is lower than 1 mol%. The longer rise times of such samples indicates that the energy transfer is the dominant mechanism for the upconversion process of the Nd^3+^–Yb^3+^–Er^3+^ system with a small amount of Nd^3+^ ions under 808-nm excitation. However, the rise time decreases from 1 to 0.4 ms as the concentration of Nd^3+^ is increased from 0.25 to 3 mol%. As the concentration of the Nd^3+^ ions is increased, the interionic distance is decreased, and hence, the transition probability increases. As a consequence, the rise time decreases with the increase of concentration, since the transfer rate is the inverse of time [23].

**Table 1:** Rise times and lifetimes for individual 540-nm upconversion emission of NaYF_4_: Yb/Er/Nd (20/2/*X* mol%) nanoparticles.

%Nd^3+^	*X* = 0	*X* = 0.25	*X* = 0.5	*X* = 1	*X* = 2	*X* = 3
rise time (μs)	724	1028	903	892	540	412
lifetime (μs)	203.6	185.6	171.6	161.8	151.1	150.6

**Figure 7:**
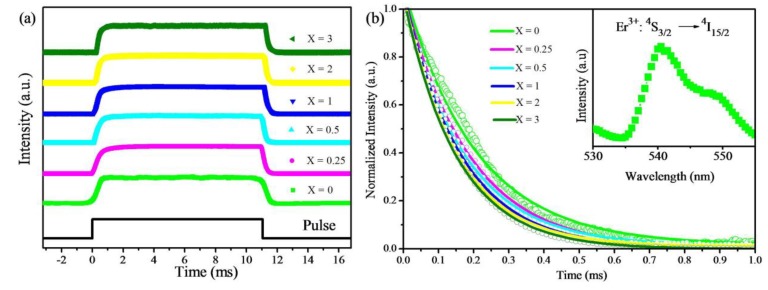
(**a**) Luminescence rise and decay curves of the Er^3+^: ^4^S_3/2_ → ^4^I_15/2_ transition in NaYF_4_: Yb/Er/Nd (20/2/*X* mol%) nanoparticles under a pulse 808-nm excitation (pulse width = 11.05 ms); (**b**) luminescence decay curves of the Er^3+^: ^4^S_3/2_ → ^4^I_15/2_ transition in NaYF_4_: Yb/Er/Nd (20/2/*X* mol%) nanoparticles. The inserted image is the emission spectrum of the ^4^S_3/2_ → ^4^I_15/2_ transition under 808-nm excitation.

The normalized decay curves of the ^4^S_3/2_ → ^4^I_15/2_ transition at 540 nm for different amounts of Nd^3+^ ions under 808-nm excitation are presented in Figure 7b. The calculated lifetimes are summarized in Table 1. The lifetime of sample NaYF_4_: Yb^3+^/Er^3+^ agrees well with the lifetime of 230 μs, which has been reported [24]. As the Nd^3+^ concentration increases, the lifetime of the ^4^S_3/2_ state decreases from 203.6 to 150.6 μs. We will discuss this trend according to the formula τ = 1/(Γ + *k*_nr_), where Γ and *k*_nr_ are the radiative and non-radiative decay rates, and τ is the lifetime [25]. The Nd^3+^ ions, having a larger ionic radius than Y^3+^ ions, are beneficial for reducing the interionic distance. Both the radiative and non-radiative decay rates of Er^3+^ ions will speed up with the decrease of the separation distance [26]. Thus, the lifetime will decrease correspondingly. However, compared to the non-radiative decay rate, the radiative decay rate increases more rapidly as the ionic separation distance decreases [27]. The radiative transition is the dominant process, which can be attributed to luminescence enhancement. The observed emission enhancement is mainly due to the competition between the changes in radiative and non-radiative decay rates. After reaching a certain distance, the higher the Nd^3+^ concentration is, the faster the energy back transfer (Er^3+^ → Nd^3+^) will be, which causes the decrease of the lifetime, and the less the luminescence enhancement or even quenching. Furthermore, the increased Nd^3+^ concentration leads to a reduced average distance between the adjacent ions, which tend to non-radiatively cross-relax (CR). The process is described as:

(^4^F_3/2_:^4^I_9/2_) → (^4^I_15/2_:^4^I_15/2_) (Δ*E* = +102 cm^−1^)

The non-radiative CR depopulates partially the excited ^4^F_3/2_ level, resulting in a simultaneous luminescence intensity and lifetime decrease with the increase of Nd^3+^ concentration [28].

**Figure 8:**
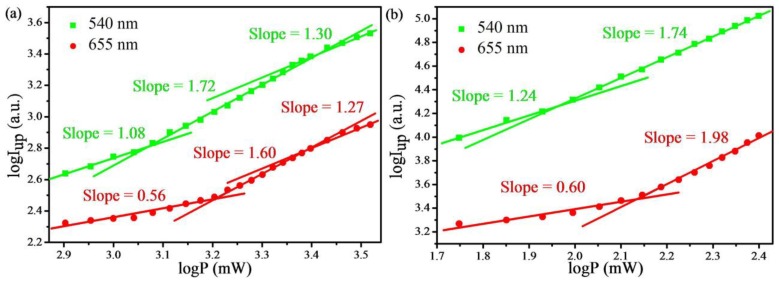
Log-log plot of the upconversion emission intensity of NaYF_4_: Yb/Er/Nd (20/2/0.5 mol%) nanoparticles as a function of the pump power under (**a**) 808-nm excitation and (**b**) 980-nm excitation.

In order to investigate the energy transfer mechanisms involved in the population of states of Er^3+^ ions, the dependence of upconversion luminescence intensity on the 808-nm and 980-nm NIR excitation power of UCNPs with *X* = 0.5 mol% as a sample is shown in Figure 8. The UC emission intensity *I*_up_ is proportional to the incident pumping power *P* as *I*_up_ ∝ *P*^n^, where *n* is the number of infrared multi-photons involved in a single visible photon generation [29]. The exponent power *n* could be determined from the slope of linear regression in a double logarithmic plot of the emitting intensity as the function of excitation intensity. The trend of the slope excited by 808-nm exactly follows that excited by 980-nm under low-power and intermediate-power pumping conditions, respectively. However, the measured slopes of the upconversion luminescence change from 1.72 to 1.30 for green fluorescence emission and from 1.60 to 1.27 for red emission in Figure 8a with increasing pump power, due to the saturation effects. The number of the luminescence center (Er^3+^) is a constant and does not change with the pump power. Therefore, the saturation effect is obviously under high power excitation. In addition, it was reported that higher pump power can increase the competition between linear decay and the upconversion process of the intermediate excited states, which results in a significantly reduced slope, too [29]. The slopes of the green fluorescence are 1.24 and 1.74, and that of the red emission are 0.60 and 1.98 under low-power and intermediate-power pumping conditions, respectively. Based on the slopes, the green and the red fluorescence emission intensity are approximately proportional to the square of the pumping power under the intermediate-power pumping condition, indicating that these emissions are performed by a two-photon process, as well as the energy loss during the upconversion process, because *n* is smaller than 2. For lower excitation powers, which are below or around an excitation power threshold, the upconverted fluorescence intensity is weak, and the crystal is nearly transparent to the pump, thus resulting in it ineffective at absorbing photons. As a result, the *n* values for the emissions are close to one under the low-power condition [30]. These values of *n* in Figure 8a are smaller than those in Figure 8b, because a photon’s energy at 808 nm is higher than that at 980 nm. It can be seen that the power threshold for 808-nm excitation is larger than that for 980 nm; because the concentration of the sensitizer Nd^3+^ ions for 808-nm excitation is much less than Yb^3+^ ions for 980-nm excitation and the Nd^3+^–Yb^3+^–Er^3+^ energy transfer may lead to more energy consumption. Furthermore, the *n* value for the green emission is larger than that for the red one excited by 808 nm, indicating a faster growth of the green fluorescence than the red one as the pump power increases. Because the *n* value for the green emission is smaller than that for the red one excited by 980 nm, which is opposite of 808 nm, it can also be proven that 808-nm excitation is favored to the green emission of Er^3+^ ions.

**Figure 9:**
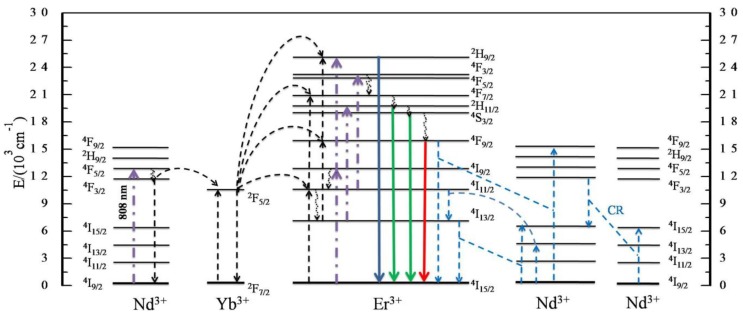
Proposed energy transfer mechanism under 808-nm excitation in NaYF_4_: Yb/Er/Nd nanoparticles. The dashed-dotted, dashed, dotted and full arrows represent photon excitation, energy transfer, multi-phonon relaxation and the emission process, respectively. For clarity, only dominant energy transfer processes are shown in the proposed mechanisms.

Having analyzed the results obtained from the upconversion emission spectra, the measurements of decay time and the dependence of luminescence intensity on excitation power, the upconversion mechanism of the Nd^3+^–Yb^3+^–Er^3+^ system under 808-nm excitation has been proposed and is shown in Figure 9. Nd^3+^ ions in the ground state are excited by an 808-nm laser and reach their excited state ^4^F_5/2_ or ^2^H_9/2_ corresponding to the transition ^4^I_9/2_ → (^4^F_5/2_, ^2^H_9/2_). The (^4^F_5/2_, ^2^H_9/2_) excited states are unstable; thus, some Nd^3+^ ions relax quickly to the ^4^F_3/2_ state through a multi-phonon non-radiative relaxation. The energy transfer (ET) between Nd^3+^ ions and Yb^3+^ ions, ^2^F_7/2_ (Yb^3+^) + ^4^F_3/2_ (Nd^3+^) → ^2^F_5/2_ (Yb^3+^) + ^4^I_11/2_ (Nd^3+^), occurs with considerably high efficiencies [31,32]. As a result, the ^2^F_5/2_ state of Yb^3+^ ions is populated. Two subsequent successive energy transfers from excited Yb^3+^ ions to Er^3+^ ions bring the Er^3+^ ions directly to their ^4^F_7/2_ state. Some Er^3+^ ions in the ^4^F_7/2_ state relax to the lower ^2^H_11/2_, ^4^S_3/2_ and ^4^F_9/2_ states through the multi-phonon relaxation, respectively; then, ^2^H_11/2_, ^4^S_3/2_ and ^4^F_9/2_ are populated. The electrons in the ^2^H_11/2_, ^4^S_3/2_ and ^4^F_9/2_ states jump to the ground state ^4^I_15/2_ and give the green (520 nm, 540 nm) and red (655 nm) emissions.

## 3. Experimental Section

### 3.1. Synthesis of Nd^3+^–Yb^3+^–Er^3+^-doped β-NaYF_4_

YCl_3_·6H_2_O (99.999%), NdCl_3_ (99.99%), YbCl_3_ (99.998%), ErCl_3_ (99.995%), NaOH, NH_4_F, oleic acid (OA), l-octadecene (ODE) and dry methanol were purchased from the Sigma-Aldrich (St. Louis, MO, USA) company and used as raw materials without any further purification. The samples were synthesized according to the molar composition of NaY_0.78−*x*_Yb_0.2_Er_0.02_Nd*_x_*F_4_ (*x* = 0, 0.25, 0.5, 1, 1.5, 2 and 3 mol%). For the synthesis of the samples, the rare earth chlorides (containing 1 mmol rare earth chlorides in total) were added to the mixture of 15 mL ODE and 6 mL OA in a two-necked flask (50 mL) and heated to 160 °C to form a transparent solution and then cooled down to room temperature. A 10-mL methanol solution containing NaOH (0.01 g) and NH_4_F (0.148 g) was slowly added into the two-necked flask and stirred for 30 min. Subsequently, the solution was slowly heated, to remove the water, oxygen and methanol at 100 °C for 30 min, and then heated to 300 °C and maintained for 1.5 h under argon atmosphere. After the solution cooled down naturally, samples were collected by centrifugation and washed with ethanol three times.

### 3.2. Characterization

The absorption of water was measured by a UV-3101PC UV-VIS-NIR scanning spectrophotometer (Shimadzu, Kyoto, Japan). The X-ray powder diffraction (XRD) analyses were performed on a Bruker D8 Advance X-ray diffractometer (Billerica, MA, USA) with Cu K_α1_ irradiation (λ = 1.54056 Å). The transmission electron microscopy (TEM) images were recorded on a JEOL JEM-1400 transmission electron microscope (Tokyo, Japan). The upconversion luminescence spectra were recorded on a SPEX Fluorescence Spectrometer Fluordlog-3 (Jobin Yvon-Spex, Paris, France) with a resolution of 0.02 nm, while the excitation sources used were an 808-nm and a 980-nm semiconductor laser (BWT Beijing Ltd., Beijing, China) with a variable excitation power density range from 100 to 3300 mW/cm^2^ and 2 to 250 mW/cm^2^, respectively. Transient decays at 540 nm were recorded using an 808-nm and a 980-nm Raman shifter pulsed laser and square-wave modulation of the electric current input to the 808-nm and 980-nm diode laser, respectively, which were ultimately recorded using a Tektronix DPO 4104 digital phosphor oscilloscope (Beaverton, OR, USA). All of the measurements were performed at room temperature.

## 4. Conclusions

In this article, Nd^3+^–Yb^3+^–Er^3+^-doped β-NaYF_4_ nanocrystals with different Nd^3+^ concentrations were synthesized, and the UC luminescence properties of the NPs have been studied under 808-nm excitation, where water has lower absorption. All of the Nd^3+^–Yb^3+^–Er^3+^-doped UCNPs have the hexagonal crystallization, the same as β-NaYF_4_, and have a uniform morphology. The average particle sizes decrease along with the increase of Nd^3+^ concentration. The upconversion luminescence spectra of NaYF_4_ nanoparticles with different dopants under 808-nm excitation proves that the Nd^3+^ ion can absorb the photons effectively, and the Yb^3+^ ion can play the role of an energy-transfer bridging ion between the Nd^3+^ ion and Er^3+^ ion. To investigate the effect of the Nd^3+^ ion, the decay curves of the ^4^S_3/2_ → ^4^I_15/2_ transition at 540 nm was measured and analyzed. The NaYF_4_: 20%Yb^3+^, 2%Er^3+^, 0.5%Nd^3+^ nanocrystals have the highest emission intensity among all of the samples under 808-nm excitation. It was concluded that the Nd^3+^ ion, with suitable content, has a positive contribution to the upconversion emissions of Er^3+^ ions. The energy back transfer from Er^3+^ ions to Nd^3+^ ions becomes more efficient when the Nd^3+^ concentration is more than 0.5 mol%. This was proven by the dependence of the upconversion intensity on the concentration of Nd^3+^ ions and the decay curves of 540-nm emission under 980-nm excitation. The upconversion mechanism under 808 nm is a two-photon process. These results make it possible to minimize the overheating effect in biological applications by widening the wavelength of the excitation source to 808 nm. We believe that such luminescent UCNPs will provide a new tool for a wide variety of applications in the fields of bioanalysis and biomedicine.

## References

[1] Mullen T.J., Zhang M., Feng W., El-khouri R.J., Sun L.D., Yan C.H., Patten T.E., Liu G. (2013). Fabrication and characterization of rare-earth-doped nanostructures on surfaces. ACS Nano.

[2] Li W., Wang J., Ren J., Qu X. (2014). Near-infrared upconversion controls photocaged cell adhesion. J. Am. Chem. Soc..

[3] Liu Y., Tu D., Zhu H., Ma E., Chen X. (2013). Lanthanide-doped luminescent nano-bioprobes: From fundamentals to biodetection. Nanoscale.

[4] Su L.T., Karuturi S.K., Luo J., Liu L., Liu X., Guo J., Sum T.C., Deng R., Fan H.J., Liu X. (2013). Photon upconversion in hetero-nanostructured photoanodes for enhanced near-infrared light harvesting. Adv. Mater..

[5] Zhou L., Li Z., Liu Z., Yin M. (2014). One-step nucleotide-programmed growth of porous upconversion nanoparticles: Application to cell labeling and drug delivery. Nanoscale.

[6] Sun Y., Zhu X., Peng J., Li F. (2013). Core-shell lanthanide upconversion nanophosphors as four-modal probes for tumor angiogenesis imaging. ACS Nano.

[7] Li C., Yang D., Ma P., Chen Y., Wu Y., Hou Z., Dai Y., Zhao J., Sui C., Lin J. (2013). Multifunctional upconversion mesoporous silica nanostructures for dual modal imaging and *in vivo* drug delivery. Small.

[8] Wong H.T., Tsang M.K., Chan C.F., Wong K.L., Fei B., Hao J. (2013). *In vitro* cell imaging using multifunctional small sized KGdF4: Yb^3+^, Er^3+^ upconverting nanoparticles synthesized by a one-pot solvothermal process. Nanoscale.

[9] Guo H., Dong N., Yin M., Zhang W., Lou L., Xia S. (2004). Visible upconversion in rare earth ion-doped Gd_2_O_3_ Nanocrystals. J. Phys. Chem. B.

[10] Auzel F.E. (1973). Materials and devices using double-pumped-phosphors with energy transfer. Proc. IEEE.

[11] Boyer J.C., Cuccia L.A., Capobianco J.A. (2007). Synthesis of colloidal upconverting NaYF_4_: Er^3+^/Yb^3+^ and Tm^3+^/Yb^3+^ monodisperse nanocrystals. Nano Lett..

[12] Zhan Q., Qian J., Liang H., Somesfalean G., Wang D., He S., Zhang Z., Andersson-Engels S. (2011). Using 915 nm laser excited Tm^3+^/Er^3+^/Ho^3+^-doped NaYbF_4_ upconversion nanoparticles for *in vitro* and deeper *in vivo* bioimaging without overheating irradiation. ACS Nano.

[13] Wang Y.F., Liu G.Y., Sun L.D., Xiao J.W., Zhou J.C., Yan C.H. (2013). Nd^3+^-sensitized upconversion nanophosphors: Efficient *in vivo* bioimaging probes with minimized heating effect. ACS Nano.

[14] Heer S., Kömpe K., Güdel H.U., Haase M. (2004). Highly efficient multicolour upconversion emission in transparent colloids of lanthanide-doped NaYF_4_ nanocrystals. Adv. Mater..

[15] Chen D., Wang Y., Yu Y., Liu F., Huang P. (2007). Sensitized thulium ultraviolet upconversion luminescence in Tm^3+^/Yb^3+^/Nd^3+^ triply doped nanoglass ceramics. Opt. Lett..

[16] Kushida T., Marcos H.M., Geusic J.E. (1986). Laser transition cross section and fluorescence branching ratio for Nd^3+^ in yttrium aluminum garnet. Appl. Phys. B.

[17] Shannon R.D. (1976). Revised effective ionic radii and systematic studies of interatomic distances in halides and chalcogenides. Acta Crystallogr. A.

[18] Meza O., Diaz-Torres L.A., Salas P., de la Rosa E., Solis D. (2010). Color tunability of the upconversion emission in Er-Yb doped the wide band gap nanophosphors ZrO_2_ and Y_2_O_3_. Mater. Sci. Eng. B.

[19] Liu Y., Wang D., Shi J., Peng Q., Li Y. (2013). Magnetic tuning of upconversion luminescence in lanthanide-doped bifunctional nanocrystals. Angew. Chem. Int. Ed..

[20] Du P.W., Eisenberg R. (2010). Energy upconversion sensitized by a platinum(II) terpyridyl acetylide complex. Chem. Sci..

[21] Xie X.J., Gao N.Y., Deng R.R., Sun Q., Xu Q.H., Liu X.G. (2013). Mechanistic investigation of photon upconversion in Nd^3+^-sensitized core-shell nanoparticles. J. Am. Chem. Soc..

[22] Zhong Y., Tian G., Gu Z., Yang Y., Gu L., Zhao Y., Ma Y., Yao J. (2014). Elimination of photon quenching by a transition layer to fabricate a quenching-shield sandwich structure for 800 nm excited upconversion luminescence of Nd-sensitized nanoparticles. Adv. Mater..

[23] Balda R., Fernández J., Saez de Ocáriz I., Voda M., Garcia A.J. (1999). Laser spectroscopy of Pr^3+^ ions in LiKY_1−*x*_Pr*_x_*F_5_ single crystals. Phys. Rev. B.

[24] Shan J., Uddi M., Yao N., Ju Y. (2010). Anomalous raman scattering of colloidal Yb^3+^, Er^3+^ codoped NaYF_4_ nanophosphors and dynamic probing of the upconversion luminescence. Adv. Funct. Mater..

[25] Fu Y., Zhang J., Lakowicz J.R. (2009). Silver-enhanced fluorescence emission of single quantum dot nanocomposites. Chem. Commun..

[26] Yuan P., Lee Y.H., Gnanasammandhan M.K., Guan Z., Zhang Y., Xu Q.H. (2012). Plasmon enhanced upconversion luminescence of NaYF_4_: Yb,Er@SiO_2_@Ag core–shell nanocomposites for cell imaging. Nanoscale.

[27] Esteban R., Laroche M., Greffet J.J. (2009). Influence of metallic nanoparticles on upconversion processes. J. Appl. Phys..

[28] Bednarkiewicz A., Wawrzynczyk D., Nyk M., Srek W. (2011). Optically stimulated heating using Nd^3+^ doped NaYF_4_ colloidal near infrared nanophosphors. Appl. Phys. B.

[29] Pollnau M., Gamelin D.R., Luthi S.R., Gudel H.U., Hehlen M.P. (2000). Power dependence of upconversion luminescence in lanthanide and transition-metal-ion systems. Phys. Rev. B.

[30] Joubert M.F. (1999). Photon avalanche upconversion in rare earth laser materials. Opt. Mater..

[31] Weber M.J. (1971). Optical properties of Yb^3+^ and Nd^3+^–Yb^3+^ energy transfer in YAlO_3_. Phys. Rev. B.

[32] Liégard F., Doualan J.L., Moncorgé R., Bettinelli M. (2005). Nd^3+^ → Yb^3+^ energy transfer in a codopedmetaphosphate glass as a model for Yb^3+^ laser operation around 980 nm. Appl. Phys. B.

